# Medical Education

**Published:** 1903-06-20

**Authors:** 


### The Hospital.

A Journal of

The Medical Sciences and Hospital Administration.

Vol. XXXIV.—No. 873. Edited by Sir Henry Burdett, K.C.B. [June 20, 1903.

### Medical Education.

The grave questions pending between the General Medical Council and some of the London bodies which are empowered to grant medical qualifications, questions to which we have more than once drawn the attention of our readers, have become more acute since last we noticed them. At the recent session of the Council reports dealing with the nature of the preliminary scientific education accepted by the London Royal Colleges and the Society of Apothecaries, and with the quality of the examinations based upon it, were presented by the visitors appointed by the Council, Dr. Windle and Professor Campbell Brown, and their importance was felt to be too great to permit of their being considered at the fag end of an ordinary meeting. A special meeting of the Council was therefore resolved upon to assemble on July 15th, and in the meanwhile the reports have been published in the minutes of the Council, and thus rendered accessible to all who may desire to study them.

It will be remembered that the matters at issue arose out of the determination of the Council to extend the curriculum of medical study from four years to five, with the proviso that the first year might be devoted to physics and biology at any educational institution recognised by the Council. This determination, which was very generally approved by the profession, was found, after a time, to be rendered of no effect, so far as the two London Royal Colleges were concerned, by their recognition of ordinary schools as places in which the first year's study might be pursued, and complaint was made to the Council by the Royal College of Surgeons in Ireland to the effect, that their own acceptance of the new regulations served no other purpose than to handicap their own students unfairly. The Council, on inquiry, found this to be the case, and found also that the London Colleges were recognising, for the purposes of the supposed first year of medical study, institutions of which the Council itself was unable to approve. The Council thereupon declined to register as medical students the young gentlemen who were being taught at the institutions in question; and the colleges thereupon determined to admit them to all parts of the medical examinations, whether they had been registered or not. The Council and the colleges were thus brought into direct collision; and the only method of coercion open to the former was to report to the Privy Council, that the courses of study and the examinations required by the conjoint board were no longer "sufficient" to secure the possession of proper knowledge and skill by those who underwent them. Such a report would have thrown the onus of decision upon the Privy Council, and the Medical Council, whether supported or not by the superior tribunal, would at least have discharged the duty imposed upon it by statute. Before taking this course it was determined to inspect the actual examinations required by the recalcitrant bodies, and to ascertain what was expected by them from the courses of study in physics and biology which they had declared to be adequate to the wants of the future members of the medical profession. The Society of Apothecaries was found to have been pursuing the same course as the Royal Colleges, and its preliminary examination has accordingly been included in the inspection.

The results, as they are embodied in the reports now given to the public, appear to be such as fully to justify the course taken by the Medical Council in relation to the matter. The visitors declare the ignorance of most of the students presenting themselves for the examination in physics and biology of the Royal Colleges to be "unfathomable," and the examination itself to be of a very unsatisfactory character when considered in relation to its supposed objects. Of the similar examination of the Society of Apothecaries they say that the standard "is not only too low, but is of a kind that is of extremely little value," and that "the average training of the candidates is insufficient for men who are commencing medical studies." We have therefore an official declaration of the most serious and deliberate description, tending to show that the early education of medical students, as conducted under the regulations of three of the London qualifying bodies, is not of such a kind as adequately to prepare their minds for the farther work which they will immediately be called upon to undertake; or, in other words, that the schools recognised by these examining bodies send away their pupils insufficiently prepared for the commencement of definitely medical studies.

The reasons of the conditions which are thus described are not difficult of comprehension. When the Medical Act of 1858 was passed it was foreseen that it would lead to greatly increased competition between the existing licensing bodies, and a machinery by which this competition might be controlled was provided by the institution of the Medical Council, to which, however, sufficient power for the fulfilment of its duties was not entrusted by the legislature. The competition is now beginning to produce its natural effects, and the Council cannot remain indifferent to them. It has incumbent upon it the plain duty of endeavouring to secure, that the foundations of medical education are well and truly laid, and are not weakened by the desire of the qualifying bodies to build a remunerative superstructure before those foundations are complete. If, as seems probable, the ultimate decision be referred to the Privy Council, it is to be hoped that the Medical Council will receive adequate support from public and professional opinion. The matter is one as to which it is possible for many practitioners to remain indifferent, but such an attitude would be distinctly unwise. A well-founded belief in the general insufficiency of medical education would seriously lower the status of all medical practitioners, and would greatly diminish the respect entertained for their opinions.

